# Supplementary material for: Opioid Use Disorder and Alternative mRNA Splicing in Reward Circuitry
Source: Genes (Basel). 2022 Jun 10;13(6):1045. doi: 10.3390/genes13061045 (PMC9222793; doi:10.3390/genes13061045)
Supplement: Supplementary file 1 [file genes-13-01045-s001.zip › Supplementary Figures S1-S4.pdf]

Supplement

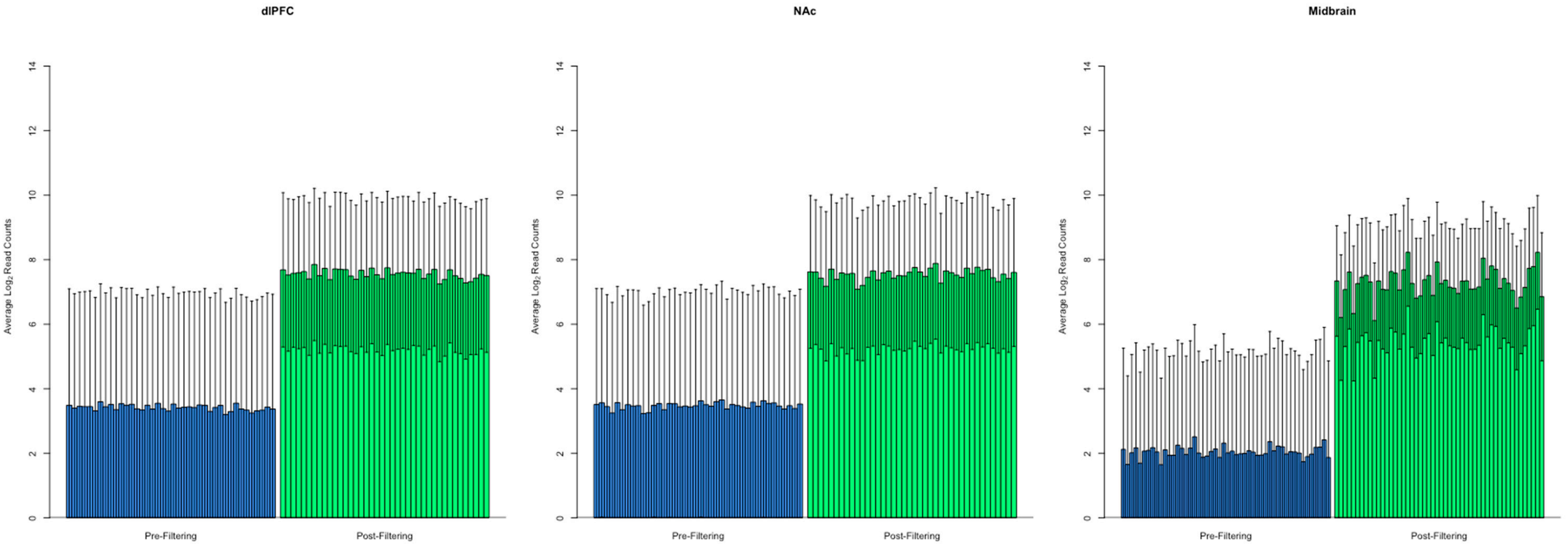

Supplementary Figure S1 RNA-seq Read Counts Across Brain Regions Before and After Filtering.

Barplot showing the low RNA-seq read counts pre-filtering. Each bar is an individual that shows the mean/SD of the RNA-seq read counts. The blue bars show the data pre-filtering and the green bars show the mean/SD RNA-seq read counts after filtering. These plots indicate good quality data and detect no outlying samples after filtering.

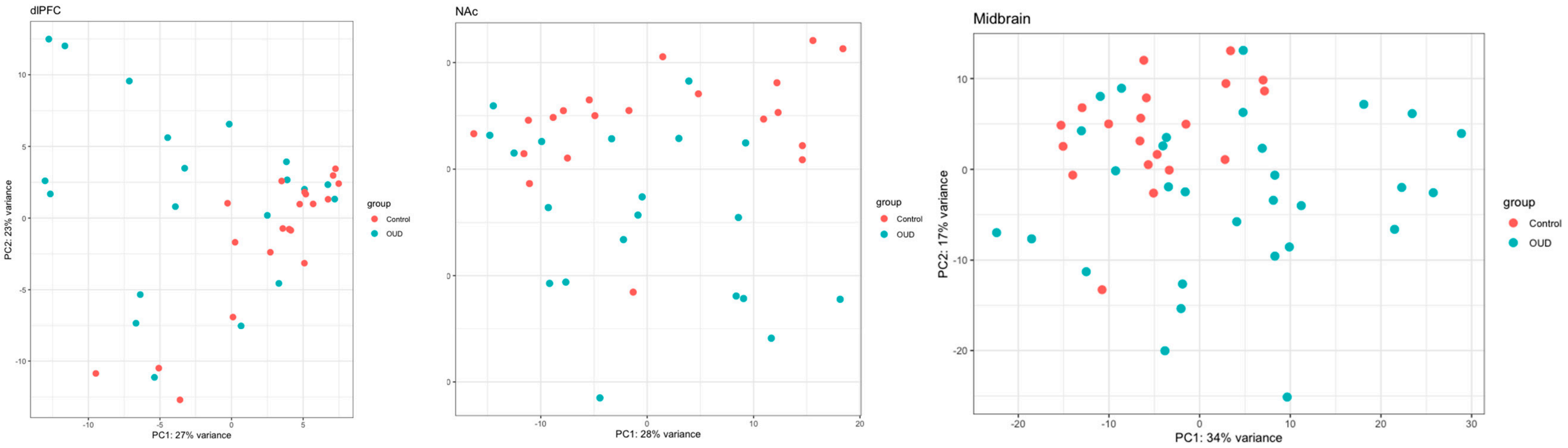

Supplementary Figure S2. Principal Components Analysis of Normalized RNA-seq Read Counts Across Brain Regions.

Scatter plots showing principal components dimensionality reduction via principal components analysis. Each dot is a sample, which are color coded by case control status.

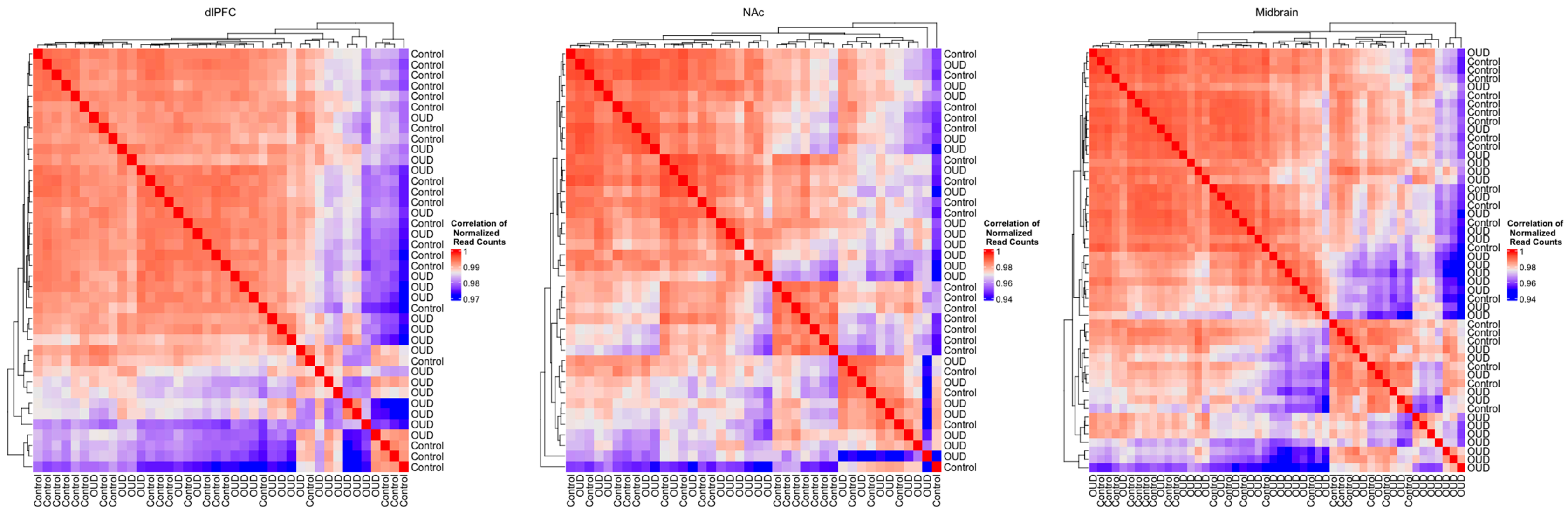

Supplementary Figure S3. Heatmap of Correlations of Normalized RNA-seq Read Counts.

Heatmaps showing association of correlation of normalized read counts across samples. Each sample was normalized via a variance-stabilized transformation and then Pearson correlation analyses were performed across all normalized genes across samples. We found a high degree of correlation across samples (all r > 0.94) indicating no major outliers in our samples.

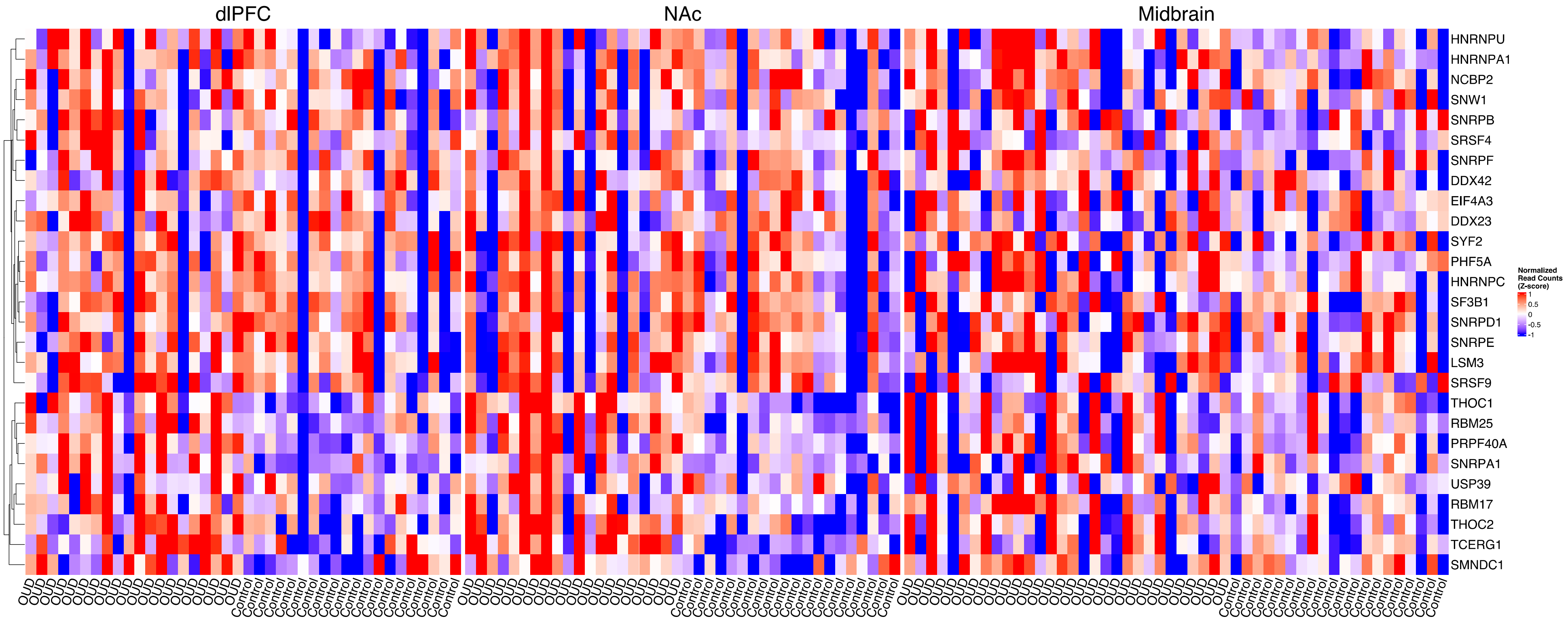

Supplementary Figure S4. Heatmaps of the 18-spliceosome genes driving enrichment across brain regions.

Heatmaps show the common “leading edge genes” of the Spliceosome KEGG pathway implicated in OUD by brain region. Z-score transformation of variance stabilized normalized RNA-seq read counts. Each column is a sample/individual and each row is a gene. Red denotes increased and blue denotes decreased expression of a gene in OUD.
